# LncRNA DDX11 antisense RNA 1 promotes EMT process of esophageal squamous cell carcinoma by sponging miR-30d-5p to regulate SNAI1/ZEB2 expression and Wnt/β-catenin pathway

**DOI:** 10.1080/21655979.2021.2008759

**Published:** 2021-12-06

**Authors:** Yanli Guo, Pingping Sun, Wei Guo, Qing Yin, Junshu Han, Supeng Sheng, Jia Liang, Zhiming Dong

**Affiliations:** a Hebei Cancer Institute, The Fourth Hospital of Hebei Medical University, Shijiazhuang, China; b Beijing Shijitan Hospital, Capital Medical University, Beijing, China

**Keywords:** Esophageal squamous cell carcinoma, DDX11-AS1, miR-30d-5p, SNAI1, ZEB2, epithelial mesenchymal transition, Wnt signaling pathway

## Abstract

LncRNA DDX11 antisense RNA 1 (DDX11-AS1) is recognized as having an imperative oncogenic role in different types of human cancer. Nevertheless, the functions, as well as the basic mechanisms of DDX11-AS1 in the EMT process of esophageal squamous cell carcinoma (ESCC), are yet to be clarified. In this research, high DDX11-AS1 expression was detected in ESCC cells as well as tissues and was linked to the poor prognosis of patients with ESCC. DDX11-AS1 promoted cell proliferation, migration, invasion ability and epithelial mesenchymal transition (EMT) process in vitro. Mechanistic analysis depicted that DDX11-AS1 may function as a ceRNA through sponging miR-30d-5p to upregulate the expression of SNAI1 and ZEB2. Meanwhile, overexpression of DDX11-AS1 might cause the activation of the Wnt/β-catenin signaling pathway via targeting miR-30d-5p. On the whole, the findings of this research illustrate that DDX11-AS1 may act as an EMT-related lncRNA to advance ESCC progression through sponging miR-30d-5p to regulate SNAI1/ZEB2 expression and activate the Wnt/β-catenin pathway, which indicates that it might serve as a probable therapeutic target for ESCC.

Esophageal cancer has been found to be among the most aggressive cancers with high mortality and poor prognosis [1]. There are two major esophageal cancer subtypes including esophageal adenocarcinoma (EAC) and esophageal squamous cell carcinoma (ESCC), and which are dissimilar in epidemiology and biology. About 70% of global esophageal cancer patients were in China, and ESCC has been found to be the main histological type of Chinese patients, responsible for 90%[2]. Despite the ongoing advancement in existing therapies, the 5-year overall survival (OS) rate of ESCC patients is still below 20% [3,4], generally ascribed to metastasis in the early stage of the tumor. Therefore, investigating the biomarkers and detecting the molecular mechanisms of metastasis can provide guidance of the therapeutic strategies and prolong survival of ESCC patients.

Growing evidence showed that dysregulated Long non-coding RNAs (lncRNAs) took part in tumor progression and metastasis via numerous mechanisms [5,6], including transcription, posttranscriptional regulation, and chromatin modification [7]. As for ESCC, a large number of lncRNAs have been reported to contribute to the initiation and development of it by our and other groups [8–10]. Epithelial-mesenchymal transition (EMT) has been recognized as being of vital importance in the initial events of tumor cells metastasis. Members of the transforming growth factor β (TGF-β) family control numerous cellular functions to modulate tumorigenesis and progression [11,12]. Emerging studies indicated that TGF-β could serve as a possible signal to instigate and drive EMT. In order to detect and illustrate novel factors that possibly related to TGF-β-induced tumor aggression in ESCC, we treated Eca109 ESCC cell line with TGF-β1 according to a previous approach [13], which caused Eca109 cells to undergo EMT. Many dysregulated lncRNAs were subsequently detected by microarray analysis. Among them, we focused on the lncRNA DDX11 antisense RNA 1 (DDX11-AS1), for its significant upregulation after TGF-β1 treatment. DDX11-AS1, an antisense lncRNA situated at chr11p14.2-p14.1, has recently been revealed to function as a novel cancer-related lncRNA to be upregulated and served as a candidate oncogenic lncRNA in various human cancer types [14–23],including esophageal carcinoma [23]. Li et al. recently reported the oncogenic function of DDX11-AS1 by targeting miR-514b-3p/RBX1 axis in esophageal carcinoma. In general, lncRNAs may operate via a variety of molecular mechanisms in the same tumor. For example, lncRNA SNHG17 regulated cell proliferation and invasion by targeting miR-338-3p/SOX4 axis in ESCC [24]. Meanwhile, it also promoted the EMT process via up-regulating the expression of c-Myc by binding to c-Jun in ESCC [25]. ESCC is prone to invasion and metastasis, and EMT plays an essential role in this process. As a marked upregulated lncRNA in TGF-β1 treated ESCC cells, the roles and fundamental mechanisms of DDX11-AS1 in the EMT process of ESCC need further verification.

Considering the significant upregulation of DDX11-AS1 in TGF-β1 treated ESCC cells, we speculated that DDX11-AS1 was involved in the EMT process of ESCC. In the present study, we detected the expression level of DDX11-AS1 in ESCC cells and tissues, as well as the EMT related markers to evaluate the effect of DDX11-AS1 on EMT process. Meanwhile, we investigated the effect of miR-30d-5p/SNAI1/ZEB2 axis and Wnt/β-catenin pathway on the DDX11-AS1 mediated EMT process. It may provide novel insights into therapeutic targets and prognostic markers for ESCC.

## Materials and methods

### Specimens and participants

The tumor tissues as well as the paired contiguous normal tissues were obtained from 144 participants with ESCC at the Fourth Affiliated Hospital, Hebei Medical University over a period of 2 years between 2012 and 2014. All the enrolled participants shared the same racial ethnicity, Han nationality, and all of them were from the same areas, which were recognized as the high-risk areas of upper gastrointestinal cancers (UGIC) in Hebei province. Individuals with at least one first-degree or two second-degree relatives with a confirmed diagnosis of gastric/cardia/esophageal cancer were considered to have a family history of UGIC. The clinicopathological information in the study cohort was illustrated in Table S1. All cases of esophageal cancers were squamous cell carcinoma while the adjacent tissues were normal tissues, which were verified by microscope examination. Survival data and Recurrence were determined via the Tumor Registry and Hospital chart review. Eight participants were lost to follow-up. The protocol of this research got approval from the ethics committee of the Fourth Affiliated Hospital of Hebei Medical University, and all the patients participating in this experiment gave their informed consent.

### Cell culture and treatment

Five human esophageal cancer cell lines (TE13, TE1, Kyse170, Eca109, and Kyse150) were grown in a controlled artificial environment with a temperature of 37°C, atmosphere consisting of 5% CO_2_ and in RPMI-1640 medium (Gibco, USA). 10 ng/ml of recombinant TGF-β1 (R&D Systems) was utilized to treat the cells for a period of 21 days with the medium replenishment occurring after every 2 days, which was performed as previously reported [13].

### Cell transfection

Designing and synthesis of the siRNAs targeting DDX11-AS1 and the over-expression plasmid pcDNA3.1-DDX11-AS1 were performed utilizing GenePharma and Sangon Biotech, sequentially. The hsa-miR-30d-5p inhibitor and mimics were bought from GenePharma (Shanghai, China). The sequences of siRNAs and miR-30d-5p inhibitor/mimics were recorded in Supplementary table S2. Cell seeding was done by spreading the cells into a 6-well plate (2 × 10^5^ cells/well) and transfection was performed utilizing FuGENE HD Transfection Reagent (Promega). The disordered siRNA (siRNA-NC) and appropriate empty vector (pcDNA3.1) was utilized as a negative control.

### RNA isolation and quantitative real-time RT-PCR assay

Trizol reagent (Invitrogen, USA) was utilized for total RNA isolation from cell and tissues lines. The synthesis of cDNA was carried out utilizing the Transcriptor First Strand cDNA Synthesis kit (Roche, Switzerland) and the cDNA from each sample was employed as a qRT-PCR template. The qRT-PCR was executed in StepOne Real-time PCR system (Applied Biosystems, USA) utilizing GoTaq®qPCR Master Mix (Promega, USA). All the primer sequences were recorded in Supplementary Table S2. The normalization of these genes expression was done with glyceraldehyde-3-phosphate dehydrogenase (GAPDH) or U6 using the 2^−ΔΔCT^ method [26]. The experiment was replicated threefold for each sample to ensure quality control.

### Cell proliferation assay

Measuring the MTS assay was performed utilizing the CellTiter96®AQ_ueous_ One Solution Cell Proliferation Assay kit (Promega) [27]. Concisely, 96-well plates were utilized for seeding 3 × 10^3^ cells. Each of these well-plates was added 20 μl MTS (500 μg/ml, Promega, USA) at 0, 24, 48, 72, and 96 hours following cell attachment, and the incubation period was 2 hours. The absorbance of each well plate was identified at a wavelength of 490 nm. These experimentations were conducted threefold.

### Wound-healing assay

An aggregate of 8 × 10^5^ cells was plated in six-well plates, and subsequently scratched with the tip of a pipette and washed with PBS to get rid of suspended cells or debris. The relative distance of the scratched area was assessed and computed [28]. Photographing of cells was done at the same field after every 24 hours. These experimentations were replicated threefold.

### Cell invasion assay

The cells were suspended in a medium that was serum-free and placed into the upper chambers, which were layered with 30 μg Matrigel (BD Biosciences, USA), while 600 μl of RPMI-1640 consisting of 10% FBS was utilized to supplement the bottom chambers. Following 24 hours of incubation, migrated cells were fixed utilizing 4% paraformaldehyde and then stained utilizing crystal violet (0.5%). Lastly, cells from 5 randomly selected fields were tallied under a light microscope [27]. Each experiment was carried out threefold.

### Subcellular fractionation

In order to ascertain the cellular localization of DDX11-AS1, isolation of nuclear and cytoplasmic RNAs from cells was carried out utilizing the Nuclear/Cytosol Fractionation Kit (BioVision, USA). The isolated RNAs were exposed to qRT-PCR analysis to substantiate the cellular localization of DDX11-AS1 with GAPDH utilized for cytoplasm control and U6 utilized for nucleus control [27].

### Luciferase reporter assay

The wild-type and mutant fragments of DDX11-AS1 containing the miR-30d-5p recognition site were subcloned into pmirGLO vector (Promega) to construct pmirGLO-DDX11-AS1 (WT) or pmirGLO-DDX11-AS1 (MUT). Likewise, the 3ʹUTR sequences of SNAI1/ZEB2 containing predicated or mutated miR-30d-5p binding sites were used to synthesize pmirGLO-SNAI1/ZEB2 (WT) or pmirGLO-SNAI1/ZEB2 (MUT) vectors. Cells were co-transfected with miR-30d-5p mimics/inhibitor or negative control and corresponding reporter plasmids by lipofectamine-mediated gene transfer. To measure Wnt pathway reporter activity, cells were transfected with TOP/FOP Flash plasmid (Millipore Corporation), along with an internal Renilla control plasmid. The relative luciferase activity was measured by Dual-Luciferase Reporter Assay System (Promega, USA) and normalized to Renilla luciferase activity 48 h following transfection. The recounted data denote the average of 3 independent transfection experimentations [27,29].

### RNA immunoprecipitation (RIP) assay

The Magna RIP RNA-Binding Protein Immunoprecipitation Kit (Millipore, USA) was used for MS2-RIP assay. Subcloning of the MS2-12X fragment from pSL-MS2-12X plasmid (Addgene, USA), gave pcDNA3.1, pcDNA3.1-DDX11-AS1, pcDNA3.1-DDX11-AS1-MUT (miR-30d-5p) to build the pcDNA3.1-MS2, pcDNA3.1-MS2-DDX11-AS1, and pcDNA3.1-MS2-DDX11-AS1-MUT plasmids, in that order. Synthesis of the point mutations of DDX11-AS1 binding to miR-30d-5p was done utilizing a Q5® Site-Directed Mutagenesis kit (NEB, USA). Eca109 cells co-transfection was done with pcDNA3.1-MS2, pcDNA3.1-MS2-DDX11-AS1, pcDNA3.1-MS2-DDX11-AS1 -MUT, and pMS2-GFP (Addgene, USA) for a duration of 48 hours. Subsequently, Eca109 cells were rinsed in pre-cold PBS and lysed in RIP buffer at a temperature of 4°C for a duration of 1 hour. Treatment of cell lysates was done with normal mouse IgG or magnetic beads conjugated to GFP antibody (Roche, Switzerland) and incubated overnight. Subsequently, following RNA purification, the immunoprecipitated RNA was identified utilizing the qRT-PCR method to measure the existence of the binding targets [27].

### Bioinformatics analysis

GEPIA (http://gepia.cancer-pku.cn/about.html) is the online tool for analyzing the RNA sequencing expression data from the TCGA and the GTEx projects [30]. Starbase 3.0 (https://starbase.sysu.edu.cn/) provides comprehensive Pan-Cancer Networks of lncRNAs and miRNAs by analyzing their expression profiles across 32 cancer types integrated from TCGA project [31]. We use GEPIA or Starbase 3.0 to analyze the expression of DDX11-AS1 or miR-30d-5p in esophageal cancer tissues. The LncBase database (http://carolina.imis.athena-innovation.gr/diana_tools/web/index.php?r=lncbasev2%2Findex) was used to predict the miRNAs binding to DDX11-AS1 [32]. The target genes of miR-30d-5p were predicted by microT-CDS (http://diana.imis.athena-innovation.gr/DianaTools/index.php?r=microTCDS/index), miRWalk (http://mirwalk.umm.uni-heidelberg.de/) and miRDB (http://mirdb.org/faq.html) online tools [33,34].

### Statistical analysis

Statistical analysis was carried out utilizing the SPSS19.0 software package (SPSS Company, USA). The results of the qRT-PCR were articulated as the mean ± standard deviation (SD) and the t-test was utilized for comparing the means (all the data for comparing mean tissue expression values were statistically analyzed by paired t-test). Kaplan–Meier survival curves were charted and the Log-rank tests were utilized as required for the univariate assessment of the DDX11-AS1 expression categories. The results of the Vitro experimentations were evaluated utilizing the Student’s t-test. Two-sided tests were employed to discover significance and P values<0.05 were judged as statistically significant in all statistic tests.

## Results

In our study, we explored the biological roles and the potential molecular mechanisms of DDX11-AS1 in EMT process of ESCC. Our data showed that DDX11-AS1 was upregulated in ESCC cells and tissues. The high expression of DDX11-AS1 in tumor tissues was strongly correlated to belligerent clinicopathological features and was associated with the poor prognosis of ESCC patients. Meanwhile, over-expression of DDX11-AS1 promoted the invasion, migration, and proliferation abilities of ESCC cells. DDX11-AS1 could positively regulate the TGF-β1-induced EMT process via sponging miR-30d-5p to upregulate the expression of SNAI1/ZEB2. As the inhibitory regulators of E-cadherin, increased expression of SNAI1 and ZEB2 could further reduce the expression of E-cadherin to enhance the activity of Wnt/β-catenin pathway. DDX11-AS1 may function as an EMT-related lncRNA to promote the EMT and malignant process of ESCC.

### Increased expression of DDX11-AS1 in esophageal cancer cells and ESCC tissues

**Figure 1.**
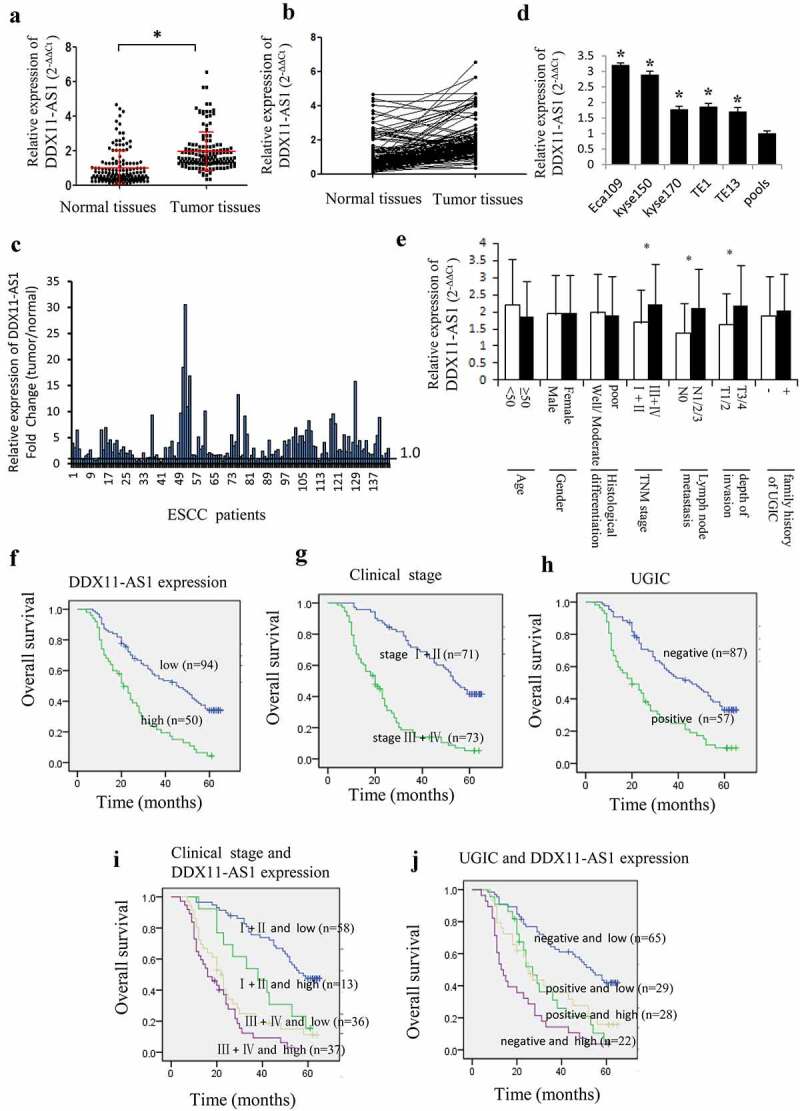
DDX11-AS1 was up-regulated in ESCC tissues and linked to poor prognosis of ESCC patients. (a) DDX11-AS1 expression was notably elevated in ESCC tissues as opposed to normal tissues, *P < 0.05. (b) DDX11-AS1 expression in tumor and matched contiguous normal tissue of each ESCC patient. (c) The fold change of DDX11-AS1 expression in tumor tissues contrasted with paired adjacent normal tissues. (d) Relative DDX11-AS1 expression level in five human esophageal cancer cell lines identified by RT-qPCR method. Pools: average expression in 10 normal tissues was utilized as normal control. (e) Correlation of the expression level of DDX11-AS1 with clinicopathologic features of ESCC patients. (f-j) Kaplan–Meier univariate survival analysis of DDX11-AS1 and TNM stage, UGIC family history in ESCC cases

The NCBI and GEPIA [30] database displayed a relatively low expression level of DDX11-AS1 in esophageal normal tissues (FigureS1A) and significant up-regulation in esophageal cancer tissues (Fig. S1B). The DDX11-AS1 expression level was corroborated by the qRT-PCR method and established to be considerably elevated in 144 ESCC tissues as opposed to matched normal tissues (P < 0.05, Figure 1(a,b)). Contrasted with normal tissues, the DDX11-AS1 expression level in 124 (86.1%) tumor tissues was up-regulated (Figure 1(c)). Among them, the expression level of DDX11-AS1 was up-regulated by more than 2 times in 84 cases, and more than 10 times in 8 cases (Figure 1(c)). Additionally, a considerably elevated expression level of DDX11-AS1 was observed in five ESCC cells, especially in Eca109 and Kyse150 cells (Figure 1(d)). Notably, when graded for clinicopathologic attributes, the DDX11-AS1 expression level was linked to TNM stage, depth of invasion, as well as lymph node metastasis (P < 0.05, Figure 1(e)).

### Up-regulated DDX11-AS1 was linked to poor prognosis of ESCC patients

The association between the expression of DDX11-AS1 and the OS of ESCC patients was evaluated utilizing the Kaplan-Meier method. The participants were categorized into relative low-expression and high-expression cohorts premised on the median DDX11-AS1 expression level. The outcomes illustrated that the OS of ESCC patients with elevated DDX11-AS1 expression levels was considerably lower as opposed to the OS of those with low expression levels (log-rank test, P < 0.05, figure 1(f)). Additionally, ESCC patients in TNM stage III and IV or with positive UGIC family history demonstrated poorer survival rate (Figure 1(g,h)). Meanwhile, ESCC cases in stage III/IV and with high expression level of DDX11-AS1 or ESCC cases with positive UGIC family history and DDX11-AS1 high expression level showed the poorest survival (log-rank test, P < 0.05, Figure 1(i,j)). In addition, univariate and multivariate analysis (Cox’s test) further demonstrated that up-regulated expression of DDX11-AS1 was an independent prognostic factor for ESCC patients’ survival (P < 0.05, Tables 1–2).

**Table 1.** Univariate analysis for OS in ESCC cases (Cox’s test)

Variable	B	SE	P	Odds ratio (95%CI)
Age	−0.246	0.214	0.250	0.782(0.515–1.189)
Gender	−0.061	0.199	0.758	0.941(0.637–1.388)
Histological grade	0.432	0.195	0.027*	1.540(1.050–2.258)
TNM stage	1.468	0.211	0.000*	4.341(2.871–6.562)
DDX11-AS1 expression	0.991	0.201	0.000*	2.695(1.816–4.000)
UGIC	0.894	0.198	0.000*	2.444(1.658–3.601)

*P < 0.05

**Table 2.** Multivariate analysis for OS in ESCC cases (Cox’s test)

Variable	B	SE	P	Odds ratio (95%CI)
Histological grade	0.526	0.198	0.008*	1.692 (1.149–2.492)
TNM stage	1.328	.228	0.000*	3.772 (2.415–5.892)
DDX11-AS1 expression	0.487	0.216	0.024*	1.627 (1.065–2.484)
UGIC	0.620	0.206	0.003*	1.858 (1.240–2.784)

*P < 0.05

### DDX11-AS1 promoted esophageal cancer cell proliferation, migration, and invasion

**Figure 2.**
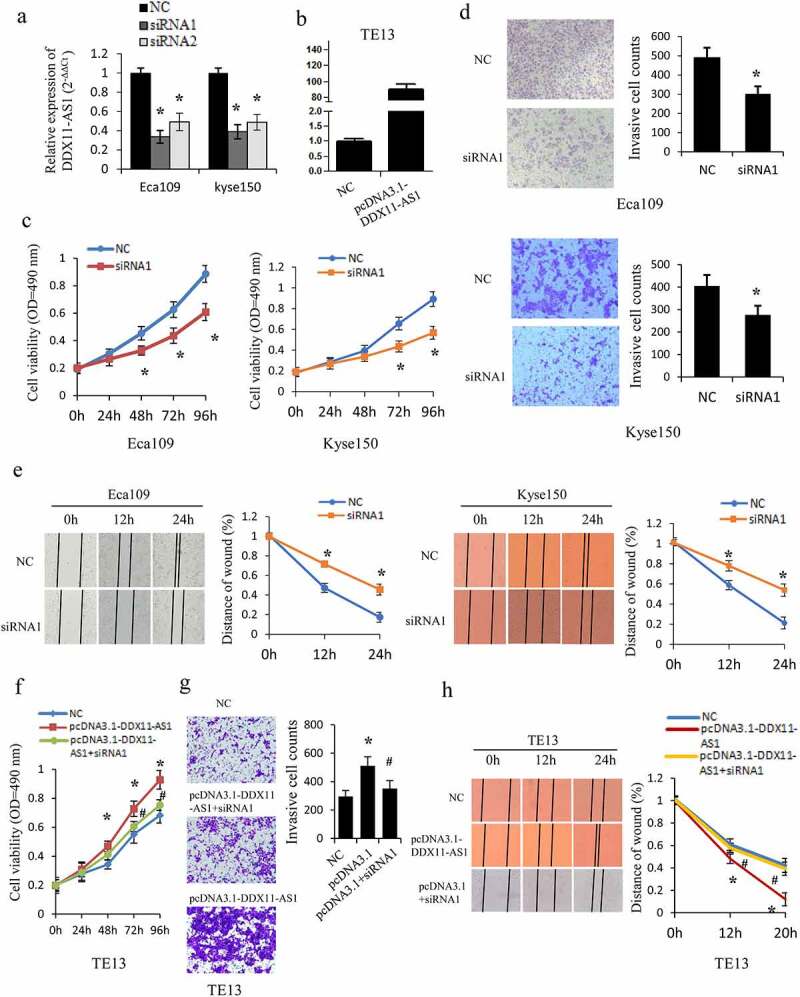
DDX11-AS1 promotes esophageal cancer cells proliferation, migration, and invasion. (a, b) The overexpression efficiency or knockdown of DDX11-AS1 transfect in esophageal cancer cells was identified by the qRT-PCR method. NC, negative control. (c) The Eca109 and Kyse150 cell growth abilities were identified by MTS. *P < 0.05, contrasted with NC cohort. (d, e) The impact of DDX11-AS1 on cell migration and invasiveness ability of Eca109 and Kyse150 cells were identified by transwell invasion assay and wound-healing experiment. *P < 0.05, contrasted with NC cohort. (f-h) The rescue experiments were performed to evaluate the function of DDX11-AS1 in TE13 cells. *P < 0.05, contrasted with NC cohort. #P < 0.05, contrasted with pcDNA3.1-DDX11-AS1 group

Considering that DDX11-AS1 expression was linked to the progression of ESCC, the gain- and loss-of-function assays were executed to examine the DDX11-AS1 biological function in vitro (Figure 2(a,b)). The siRNA-mediated knockdown of DDX11-AS1 considerably repressed the proliferation ability of Eca109 and Kyse150 cells as opposed to control cells (P < 0.05, Figure 2(c)). Meanwhile, the ability of cell invasion (P < 0.05, Figure 2(d)) and migration (P < 0.05, Figure 2(e)) were also considerably decreased after the knockdown of DDX11-AS1. Contrarily, overexpression of DDX11-AS1 markedly improved cells proliferation ability (P < 0.05, figure 2(f)), and considerably stimulated migration and invasion ability in TE13 cells (P < 0.05, Figure 2(g,h)). To further confirm the function of DDX11-AS1, rescue experiments were performed and knockdown of DDX11-AS1 was found to significantly reverse the promoted effect of DDX11-AS1 overexpression-mediated growth, migration, and invasion (P < 0.05, Figure 2(g,h)).

### DDX11-AS1 participates in TGF-β1 induced EMT process

**Figure 3.**
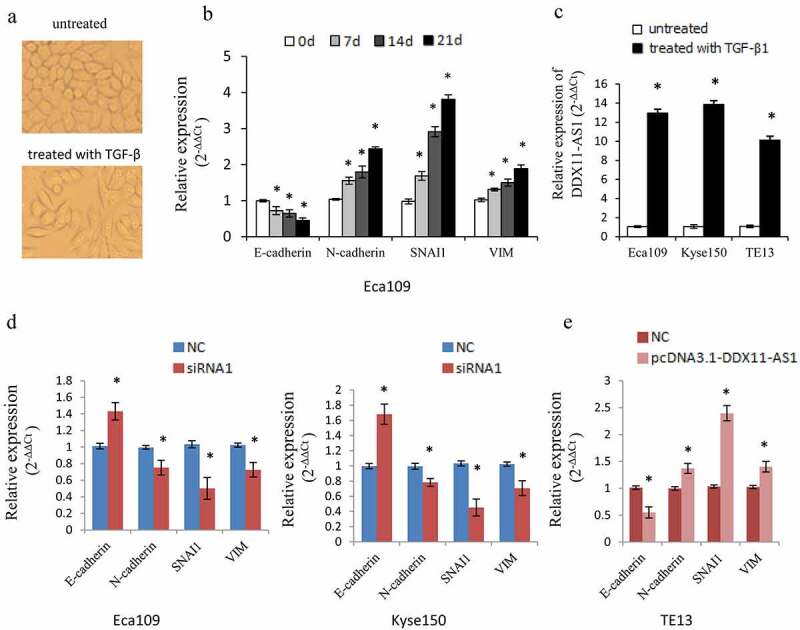
DDX11-AS1 promotes the TGF-β induced EMT process. (a) Cell morphology of Eca109 cells treated or untreated with TGF‑β. (b) Relative expression of EMT-related markers was detected in Eca109 cells with or without TGF‑β treatment. *P < 0.05, contrasted with untreated cohort. (c) Relative expression of DDX11-AS1 was identified in esophageal cancer cells with or without TGF‑β treatment. *P < 0.05, contrasted with the untreated cohort. (d, e) The effect of DDX11-AS1 on the expression of EMT-related markers. *P < 0.05, compared with NC cohort

We treated Eca109 cells continuously with TGF-β1 according to the previous approach [13]. As a result, the cells displayed morphological changes to a spindle-shaped appearance and the epithelial marker (E-cadherin) was down-regulated while the mesenchymal markers (VIM, N-cadherin, and SNAI1) were up-regulated, indicating that the cells displayed an appropriate biological response to treatment with TGF-β1 and undergoing the EMT process (Figure 3(a,b)). Meanwhile, the DDX11-AS1 expression level was considerably increased ensuing TGF-β1 treatment in Eca109, Kyse150, TE13 cells (Figure 3(c)), which indicated that DDX11-AS1 may take part in the EMT process as a downstream effector gene of TGF-β signal.

To further elucidate the function of DDX11-AS1 in the EMT process, we then identified the influence of DDX11-AS1 on EMT-associated genes. DDX11-AS1 knockdown resulted in an elevated E-cadherin expression level and reduced expression level of VIM, SNAI1, and N-cadherin (Figure 3(d)). In contrast, over-expression of DDX11-AS1 lowered the E-cadherin expression level but led to a higher expression level of VIM, N-cadherin, and SNAI1 (Figure 3(e)). These data proposed that DDX11-AS1 might be an EMT-related lncRNA and is involved in the EMT process of ESCC.

### DDX11-AS1 competitively bound to miR-30d-5p and decreases its expression in esophageal cancer cells

**Figure 4.**
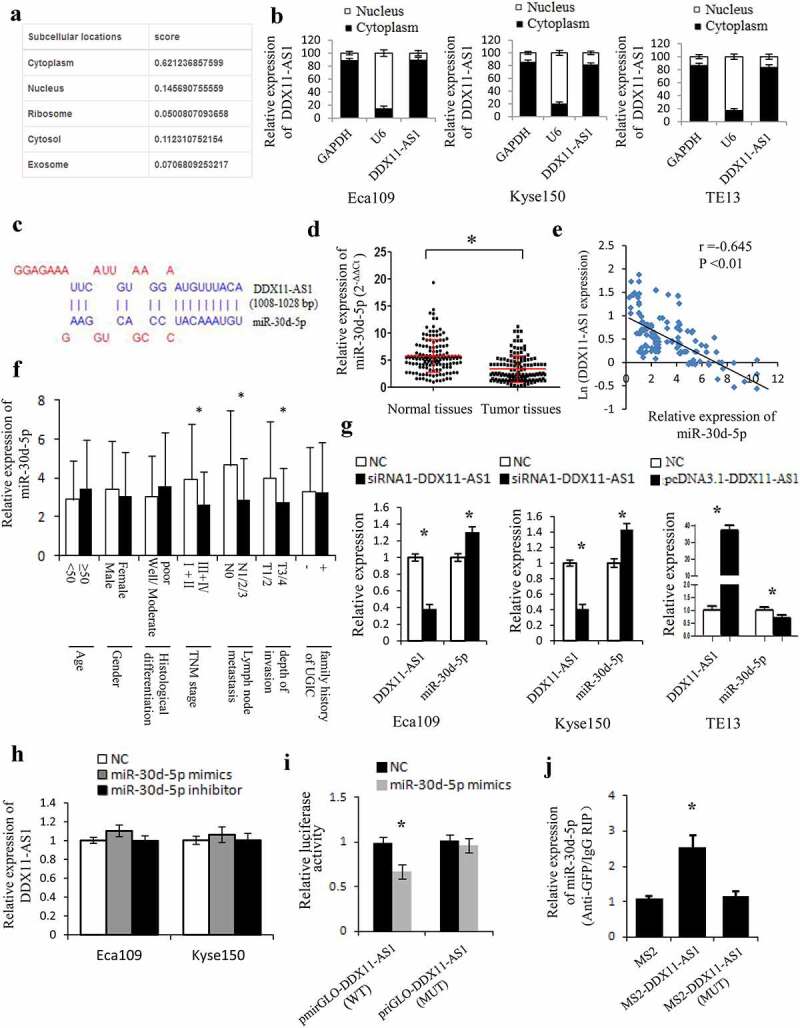
DDX11-AS1 served as a molecular sponge of miR-30d-5p. (a) The subcellular localization of DDX11-AS1 was forecasted by the lncLocator online tool. (b) The subcellular localization of DDX11-AS1 in ESCC cells was identified by the RT-qPCR method. (c) The probable binding sites of DDX11-AS1 to miR-30d-5p were forecasted by LncBase. (d) The miR-30d-5p expression was considerably reduced in ESCC tissues contrasted with normal tissues, *P < 0.05. (e) The relationship between DDX11-AS1 and miR-30d-5p expression was assessed in ESCC tissues. (f) Relative expression of miR-30d-5p in various subgroups. (g) The regulation of DDX11-AS1 on miR-30d-5p expression was identified by the qRT-PCR method. (h) MiR-30d-5p mimic or inhibitor did not affect DDX11-AS1 expression in ESCC cells. (i) The effect of miR-30d-5p mimic on the activity of DDX11-AS1 conveyed by dual-luciferase reporter assay. (j) RNA immunoprecipitation (RIP) assay illustrating the greater enrichment of miR-30d-5p to DDX11-AS1. *P < 0.05, contrasted with NC cohort

To ascertain the molecular mechanisms of DDX11-AS1 in promoting the proliferation, invasion, and EMT process of ESCC cells, the subcellular localization of DDX11-AS1 was assessed. DDX11-AS1 was primarily situated in the cytoplasm rather than in the nucleus of ESCC cells, which was consistent with the prediction of the lncLocator online tool (Figure 4(a,b)). Compelling evidences have proven that biological function of lncRNAs depends on their subcellular localization [35]. LncRNAs located in the cytoplasm could act as competing endogenous RNAs (ceRNAs) and regulate the expression of miRNA targets [36]. Therefore, we further explored the possibility that DDX11-AS1 promotes the progression of ESCC via ceRNA manner. We first predicted the probable target miRNAs of DDX11-AS1 by online bioinformatics analysis (LncBase) [32] and screened out the candidate miRNAs with higher scores (>0.8). Among these miRNAs, miR-30d-5p attracted our attention for its tumor-suppressive function in a variety of tumors [37–40]. As presented in Figure 4(c), a potential binding site was predicted between miR-30d-5p and DDX11-AS1 transcript. The remarkably reduced miR-30d-5p expression level was found in 144 ESCC tissues (P < 0.01, Figure 4(d), S1C) and was further verified by the Starbase V3.0 online tool [31] (P < 0.01, Fig. S1D). Additionally, the miR-30d-5p expression level was strongly linked to tumor invasion depth, TNM stage, and lymph node metastasis. A significant inverse relationship between miR-30d-5p and DDX11-AS1 expression level was found in ESCC tissues (P < 0.05, Figure 4(e,f)). To continue exploring the biological role of miR-30d-5p in esophageal cancer cells, we carried out the MTS and transwell assays. Upregulated miR-30d-5p led to a considerable suppression of proliferation and invasion ability in Eca109 and Kyse150 cells, whereas down-regulated miR-30d-5p displayed opposite effects (P < 0.05, Fig. S1E, 1 F), indicating that miR-30d-5p may function as a tumor suppressor in ESCC.

The association between DDX11-AS1 and miR-30d-5p was further clarified. Knocking down DDX11-AS1 leads to an elevated miR-30d-5p expression level in both Eca109 and Kyse150 cells, and overexpression of DDX11-AS1 noticeably reduced miR-30d-5p expression level in TE13 cells (P < 0.05, Figure 4(g)). Nevertheless, no significant effect on DDX11-AS1 expression level was observed after overexpression or knocking down of miR-30d-5p (Figure 4(h)). The luciferase activity of the binding domain between DDX11-AS1 wild-type (WT) and miR-30d-5p was suppressed by mimics transfection, while that between DDX11-AS1 mutant-type (MUT) and miR-30d-5p remained unchanged, signifying the binding of miR-30d-5p to DDX11-AS1 (P < 0.05, Figure 4(i)). Meanwhile, the RIP assay was executed to pull down endogenous miRNAs linked to DDX11-AS1, and miR-30d-5p was detected to be enriched by pcDNA3.1-MS2-DDX11-AS1 beads, which was not the case with pcDNA3.1-MS2-DDX11-AS1-MUT (Figure 4(j)), continuing supporting the binding ability of DDX11-AS1 to miR-30d-5p.

### SNAI1 and ZEB2, the target gene of miR-30d-5p, is regulated by DDX11-AS1

**Figure 5.**
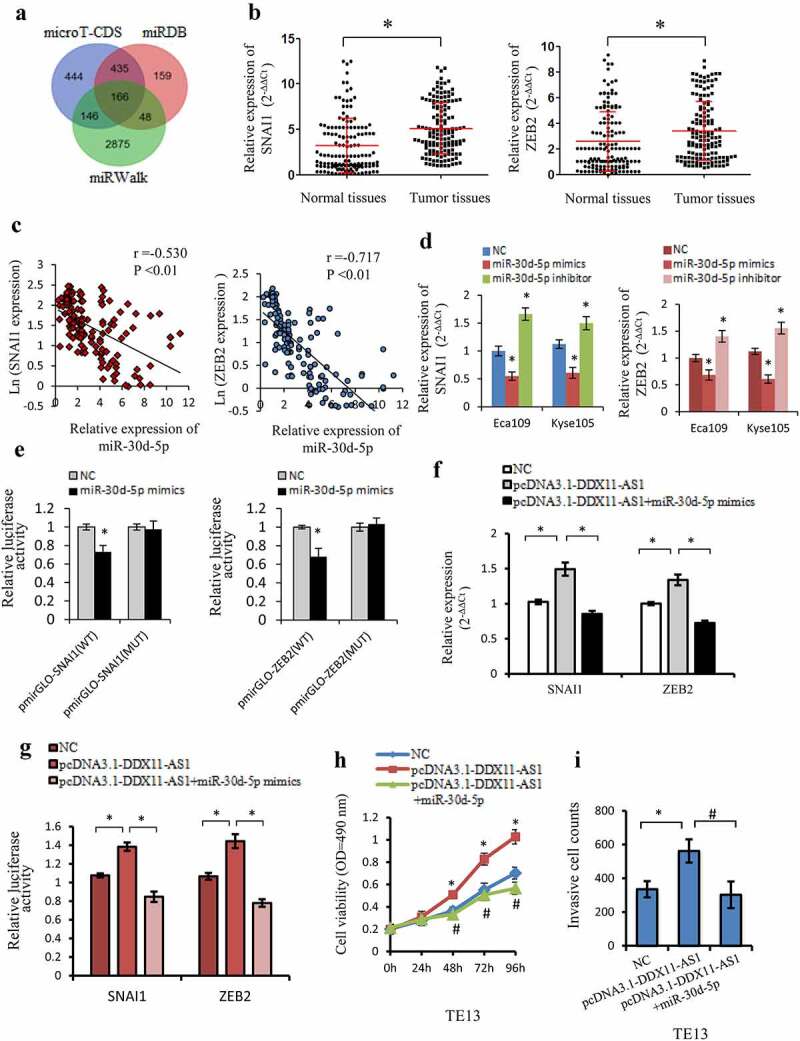
SNAI1 and ZEB2 were the target gene of miR-30d-5p and regulated by DDX11-AS1. (a) The probable target genes of miR-30d-5p are forecasted by miRDB, miRWalk v3.0, and microT-CDS. (b) SNAI1 and ZEB2 expression were considerably elevated in ESCC tissues contrasted with normal tissues, which were measured by qRT-PCR. *P < 0.05. (c) The relationship between SNAI1/ZEB2 and miR-30d-5p expression was assessed in ESCC tissues. (d) The effect of miR-30d-5p mimic/inhibitor on the expression of SNAI1/ZEB2 was reported by qRT-PCR. *P < 0.05, contrasted with NC cohort. (e) The effect of miR-30d-5p mimic/inhibitor on luciferase activity of SNAI1/ZEB2 reported by dual-luciferase reporter assay. *P < 0.05, contrasted with NC cohort. (f) The expression of SNAI1/ZEB2 was upregulated by DDX11-AS1, whereas miR-30d-5p partially rescued the increased effect in ESCC cells. *P < 0.05. (g) MiR-30d-5p partially rescued the upregulation of luciferase activity caused by DDX11-AS1 overexpression. *P < 0.05. (h, i) Cell viability and invasive cell counts were rescued by cotransfection with miR-30d-5p mimics in DDX11-AS1 overexpression cells, which were performed by MTS and transwell invasion assays. *P < 0.05, contrasted with NC cohort, #P < 0.05, contrasted with pcDNA3.1-DDX11-AS1 cohort

MiRNAs could act as regulators in numerous biological functions via suppression of the target genes expression. Several bioinformatics databases (miRWalk, microT-CDS, and miRDB) [33,34] were additionally utilized to forecast miR-30d-5p probable target genes. The target genes exhibiting a higher score (>0.8) of each prediction set were selected. Ultimately, an aggregate of 166 genes was concurrently detected in the three predictions sets (Figure 5(a)). Among them, SNAI1 and ZEB2 were well-known mesenchymal markers and involved in the regulation of EMT-related genes as transcription factors [41,42]. The binding sites of SNAI1 and ZEB2 for miR-30d-5p were illustrated in FigureS2A. The SNAI1 and ZEB2 expression levels were considerably increased in ESCC tissues, as opposed to paired adjacent normal tissues (Figure 5(b), S2B), and had a negative correlation to the expression of miR-30d-5p (Figure 5(c)). Notably, the expression level of SNAI1 and ZEB2 were all linked to TNM stage, cell invasion depth as well as lymph node metastasis (P < 0.05, Fig. S2C, 2D). Meanwhile, SNAI1 and ZEB2 expression level was elevated by miR-30d-5p inhibitor transfection, whereas reduced by miR-30d-5p mimics transfection comparing to the control cells (Figure 5(d)). The Luciferase assay further confirmed that miR-30d-5p overexpression lowered the luciferase activity of the wild-type SNAI1 and ZEB2 reporter, which was not the case with the mutant reporter (Figure 5(e)), demonstrating that SNAI1 and ZEB2 were both direct target genes of miR-30d-5p.

To better ascertain the ceRNA function of DDX11-AS1, the rescue experiments and luciferase reporter assay was executed. Overexpression of miR-30d-5p could counteract the corresponding upregulated expression and luciferase activity of SNAI1/ZEB2 induced by DDX11-AS1 overexpression in esophageal cancer cells (figure 5(f,g)). Furthermore, overexpression of DDX11-AS1 could lead to markedly improved proliferation and invasion ability in Eca109 and Kyse150 cells, but partly rescued by miR-30d-5p mimics transfection (Figure 5(h,i)). All the results illustrated that DDX11-AS1 might function as ceRNA via miR-30d-5p/SNAI1/ZEB2 axis.

### DDX11-AS1 activated the Wnt/β-catenin Signaling pathway via targeting miR-30d-5p

**Figure 6.**
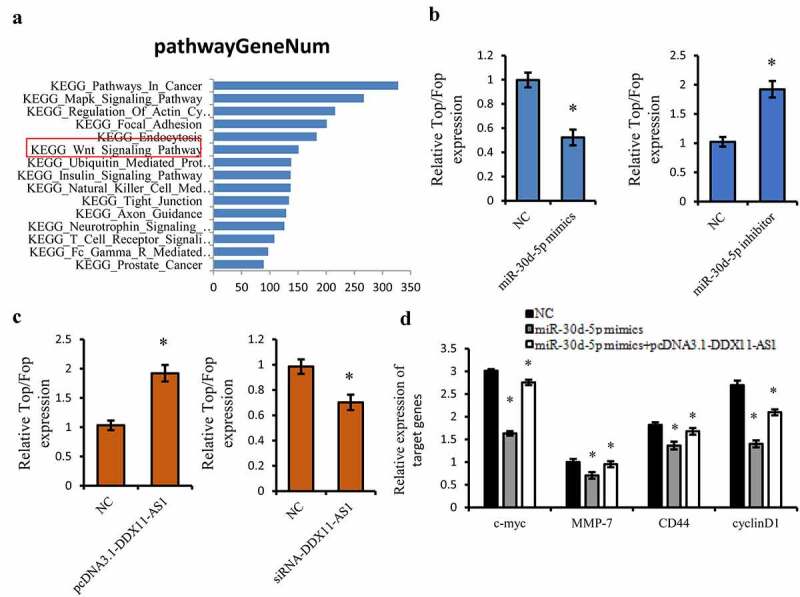
DDX11-AS1 activated the Wnt/β-catenin Signaling pathway via targeting miR-30d-5p. (a) The KEGG pathway analysis on the downstream target genes of miR-30d-5p using Starbase 3.0 online tool. (b, c) The effect of miR-30d-5p or DDX11-AS1 on the TOP/FOP Flash luciferase activity. *P < 0.05. (d) The effect of miR-30d-5p or DDX11-AS1 on downstream target genes of the Wnt pathway. *P < 0.05, contrasted with NC cohort

Considering the tumor suppressive effect of miR-30d-5p, we further performed KEGG pathway analysis on the downstream target genes of miR-30d-5p utilizing Starbase 3.0 online tool [31], and the Wnt/β-catenin signaling pathway has fascinated us for its important function in the EMT process [43,44] (Figure 6(a)). Therefore, the TOP/FOP Flash luciferase reporter gene was utilized to identify the impact of miR-30d-5p on the activation of the Wnt/β-catening pathway. MiR-30d-5p considerably inhibited the luciferase activity of Wnt signaling reporter TOP/FOP Flash (Figure 6(b)). However, overexpression of DDX11-AS1 could lead to the marked increasing of the luciferase activity of TOP/FOP Flash (Figure 6(c)). The miR-30d-5p inhibitor and knocking down of DDX11-AS1 displayed opposite effects (Figure 6(b,c)). Moreover, we also found that the expression level of downstream target genes of the Wnt pathway, such as c-myc, MMP-7, CD44, and cyclinD1, were suppressed by miR-30d-5p, whereas overexpression of DDX11-AS1 rescued these suppressive effects (Figure 6(d)). All of these findings illustrated that DDX11-AS1 induced the activation of Wnt/β-catenin signaling through precisely targeting miR-30d-5p.

### Discussion

Although reassuring progress has been realized in understanding the molecular mechanisms of ESCC, the prognosis of ESCC patients is still unfavorable [4]. Recently, increasing evidence revealed that aberrantly expressed lncRNAs were closely related to ESCC occurrence and development [5,6]. Early metastasis is the primary contributor to the high mortality of esophageal cancer [45]. EMT has been recognized as being of vital importance in the initial stages of tumor cell metastatic dissemination [46]. The method of TGF-β-induced EMT in cancer cells has been well instituted. In this research, we observed that DDX11-AS1 could positively regulate the TGF-β1-induced EMT process, which indicated that DDX11-AS1 may participate in the EMT process of ESCC as a downstream effector gene of the TGF-β signal.

Recent studies have demonstrated that DDX11-AS1, as a novel lncRNA, played an imperative function in the progression and metastasis in multiple cancers, including glioma [47], bladder cancer [21,22], gastric cancer [17,18], hepatocellular carcinoma [15], and so on [14,19,20,23]. In the current research, the DDX11-AS1 functions, as well as molecular mechanisms in EMT process of ESCC, were further explored. The high DDX11-AS1 expression level in ESCC tumor tissues was strongly correlated to belligerent clinicopathological features of ESCC patients. In the meantime, the biological function assays additionally illustrated that DDX11-AS1 encouraged ESCC cells invasion, migration, and proliferation abilities in vitro. All the findings depicted that DDX11-AS1 may function as an oncogenic lncRNA in ESCC progression. The survival analyses further proved this conclusion.

For further clarifying the possibility of DDX11-AS1 promoting the progression of ESCC via ceRNA manner, miR-30d-5p was considered as a potential candidate target gene of DDX11-AS1 for further study. MiR-30d-5p has been previously found to be a tumor suppressor in the vast majority of tumors [37–40], especially in digestive system tumors [48,49]. In current study, the miR-30d-5p expression level was considerably lowered in ESCC cells and tissues, and was conversely associated with the DDX11-AS1 expression level. Combined with the inhibiting impact of miR-30d-5p on esophageal cancer cells proliferation and invasion ability, miR-30d-5p was confirmed to act as a tumor suppressor in ESCC. Additionally, the rescue experiments, luciferase reporter, as well as RIP assays further established that DDX11-AS1 could precisely sponge and inhibit miR-30d-5p expression, as well as participated in miR-30d-5p-involved inhibiting effects on esophageal cancer cells proliferation and invasion ability.

SNAI1 and ZEB2, as EMT-initiating transcriptional factors, could promote EMT process [46]. SNAI1 and ZEB2 might directly bind to the E-cadherin promoter region and reduce its expression [41,42]. Loss of E-cadherin is a fundamental event in EMT process. In this research, we ascertained that miR-30d-5p targets SNAI1 and ZEB2 directly. Moreover, DDX11-AS1 may possibly regulate SNAI1 and ZEB2 expression in ESCC cells through competitively sponging miR-30d-5p, which substantiated the ceRNA function of DDX11-AS1. In addition, the rescue functional assays illustrated the function of DDX11-AS1/miR-30d-5p/ SNAI1/ZEB2 axis in the EMT process and ESCC progression.

KEGG pathway analysis showed the downstream target genes of miR-30d-5p mainly enriched in the Wnt/β-catenin signaling pathway and our findings also indicated that DDX11-AS1 stimulated the activation of Wnt/β-catenin signaling through directly targeting miR-30d-5p. The canonical Wnt pathway is constitutively active in a range of tumors and contributes to an imperative role in the EMT process [43,44]. The key factor of the Wnt pathway is β-catenin, which entering the nucleus and interacting with TCF/LEF to control the transcription of downstream target genes [50]. On the other hand, β-catenin forms cadherin-catenin complex with E-cadherin to regulate adhesion [50]. Thereby, β-catenin is bound at the cell surface and sequestered from the nucleus in generally. The current results revealed that DDX11-AS1 functioned as ceRNA to up-regulate SNAI1 and ZEB2 expression via sponging miR-30d-5p. As E-cadherin regulators, SNAI1 and ZEB2 may possibly bind to the E-cadherin promoter region and reduce its expression [41,42]. Reduced E-cadherin might discharge more cell-surface-sequestrated β-catenin into the cytoplasm and promote β-catenin nuclear translocation and activate Wnt/β-catenin signaling.

## Conclusion

DDX11-AS1 may act as an EMT-related lncRNA in ESCC. Upregulated expression of DDX11-AS1 promotes the malignant phenotype of ESCC cells and is related to the poor prognosis of ESCC patients. DDX11-AS1 may promote the EMT process via sponging miR-30d-5p to regulate SNAI1/ZEB2 expression and enhance the activity of Wnt/β-catenin pathway, which further affects the malignant progression of ESCC patients.

## Supplementary Material

Supplemental Material

## Data Availability

Data and materials related to this work are available upon request.

## Highlights

1. lncRNA DDX11-AS1 promotes the TGF-β-induced EMT process.
2. DDX11-AS1 promotes the malignant process of ESCC cells via miR-30d-5p/SNAI1/ZEB2 axis.
3. DDX11-AS1 enhances the activity of Wnt/β-catenin pathway via targeting miR-30d-5p.
