# Supplementary material for: LncRNA DDX11 antisense RNA 1 promotes EMT process of esophageal squamous cell carcinoma by sponging miR-30d-5p to regulate SNAI1/ZEB2 expression and Wnt/β-catenin pathway
Source: Bioengineered. 2021 Dec 6;12(2):11425–40. doi: 10.1080/21655979.2021.2008759 (PMC8810181; doi:10.1080/21655979.2021.2008759)
Supplement: Supplemental Material [file KBIE_A_2008759_SM0472.zip › supplementary/Table S1.docx]

Table S1 Clinicopathologic characteristics of esophageal squamous cell carcinoma cases

| Parameters Groups | N (%) |
| --- | --- |
| Age |  |
| <50 | 37(25.7) |
| ≥50 | 107(74.3) |
| Gender |  |
| Male | 85(59.0) |
| Female | 59(41.0) |
|  |  |
| Histological grade |  |
| Well | 37(25.7) |
| Moderate | 48(33.3) |
| Poor | 59(41.0) |
| TNM stage |  |
| Ⅰ | 6(4.2) |
| Ⅱ | 65(45.1) |
| Ⅲ | 62(43.1) |
| Ⅳ | 11(7.6) |
| Depth of invasion |  |
| T1+T2 | 60(41.7) |
| T3+T4 | 84(58.3) |
|  |  |
| LN metastasis |  |
| - | 31(21.5) |
| + | 113(78.5) |
|  |  |
| Family history of UGIC |  |
| _ | 87(60.4) |
| + | 57(39.6) |
