# Supplementary material for: LncRNA DDX11 antisense RNA 1 promotes EMT process of esophageal squamous cell carcinoma by sponging miR-30d-5p to regulate SNAI1/ZEB2 expression and Wnt/β-catenin pathway
Source: Bioengineered. 2021 Dec 6;12(2):11425–40. doi: 10.1080/21655979.2021.2008759 (PMC8810181; doi:10.1080/21655979.2021.2008759)
Supplement: Supplemental Material [file KBIE_A_2008759_SM0472.zip › supplementary/Table S2.docx]

Table S2 Primers sequences used for real-time PCR, and the sequences of siRNAs, miR-30d-5p mimics/inhibitor

| **Names** | **Sequences** |
| --- | --- |
| DDX11-AS1 | F: 5'- CAGCAACCTTTCTGGGAAGC-3' |
|  | R: 5'- ACAAGAGCTGAGCTTGTCTTT-3' |
| SNAI1 | F: 5'- ACGAGGTGTGACTAACTAT-3' |
|  | F: 5'- CGACAAGTGACAGCCATT-3' |
| ZEB2 | F: 5'-CAAGAGGCGCAAACAAGCC-3' |
|  | F: 5'-GGTTGGCAATACCGTCATCC-3' |
| E-cadherin | F: 5'-CGAGAGCTACACGTTCACGG-3' |
|  | R: 5'-GGCCTTTTGACTGTAATCACACC-3' |
| N-cadherin | F: 5'-CAACTTGCCAGAAAACTCCAGG-3' |
|  | R: 5'ATGAAACCGGGCTATCTGCTC-3' |
| VIM | F: 5'-CGCCTGCAGGATGAGATTCAG-3' |
|  | R: 5'-TCAGGGAGGAAAAGTTTGGAAGA-3' |
| c-myc | F: 5'-GGCTCCTGGCAAAAGGTCA-3' |
|  | R: 5'-CTGCGTAGTTGTGCTGATGT-3' |
| MMP7 | F: 5'-GAGTGAGCTACAGTGGGAACA-3' |
|  | R: 5'-CTATGACGCGGGAGTTTAACAT-3' |
| CD44 | F: 5'-CTGCCGCTTTGCAGGTGTA -3' |
|  | R: 5'-CATTGTGGGCAAGGTGCTATT -3' |
| cyclinD1 | F: 5'-GCTGCGAAGTGGAAACCATC-3' |
|  | R: 5'-CCTCCTTCTGCACACATTTGAA -3' |
| GAPDH | F: 5'-AGGTGAAGGTCGGAGTCAACG-3' |
|  | R: 5'-AGGGGTCATTGATGGCAACA-3' |
|  |  |
| siRNA-DDX11-AS1-1 | 5'- CTGTGTAGCTCTAGAGAAA-3' |
| siRNA-DDX11-AS1-2 | 5'- GGCCTTAAGTTTAGAGCAA-3' |
|  |  |
| Hsa-miR-30d-5p mimics | 5'- UGUAAACAUCCCCGACUGGAAG-3' |
| Hsa-miR-30d-5p inhibitor | 5'- CUUCCAGUCGGGGAUGUUUACA-3' |

F: Forward primer; R: Reverse primer
