# Supplementary figures and images for: LncRNA DDX11 antisense RNA 1 promotes EMT process of esophageal squamous cell carcinoma by sponging miR-30d-5p to regulate SNAI1/ZEB2 expression and Wnt/β-catenin pathway
Source: Bioengineered. 2021 Dec 6;12(2):11425–40. doi: 10.1080/21655979.2021.2008759 (PMC8810181; doi:10.1080/21655979.2021.2008759)

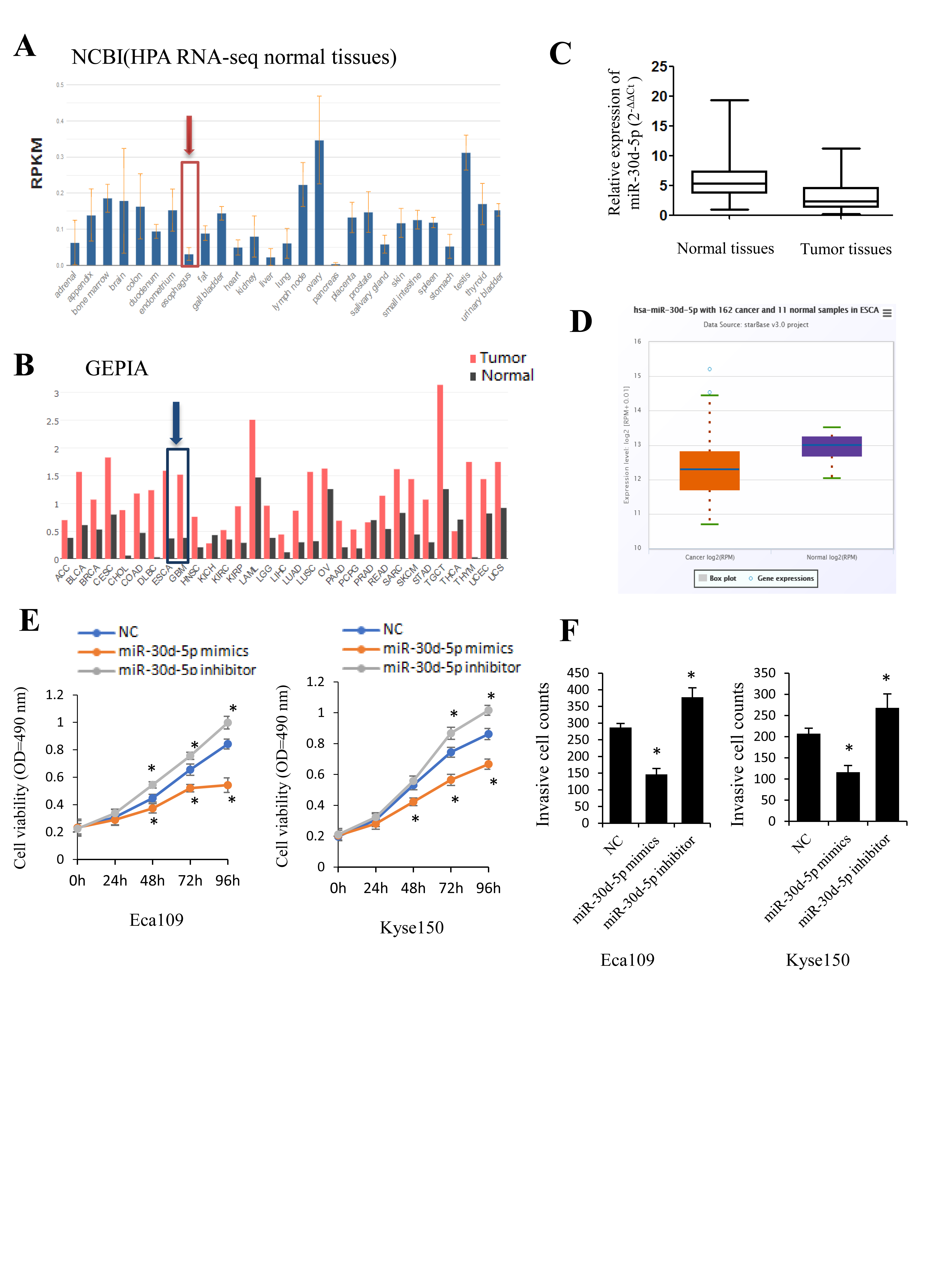

Supplement: Supplemental Material [file KBIE_A_2008759_SM0472.zip › supplementary/Fig.S1.tif]

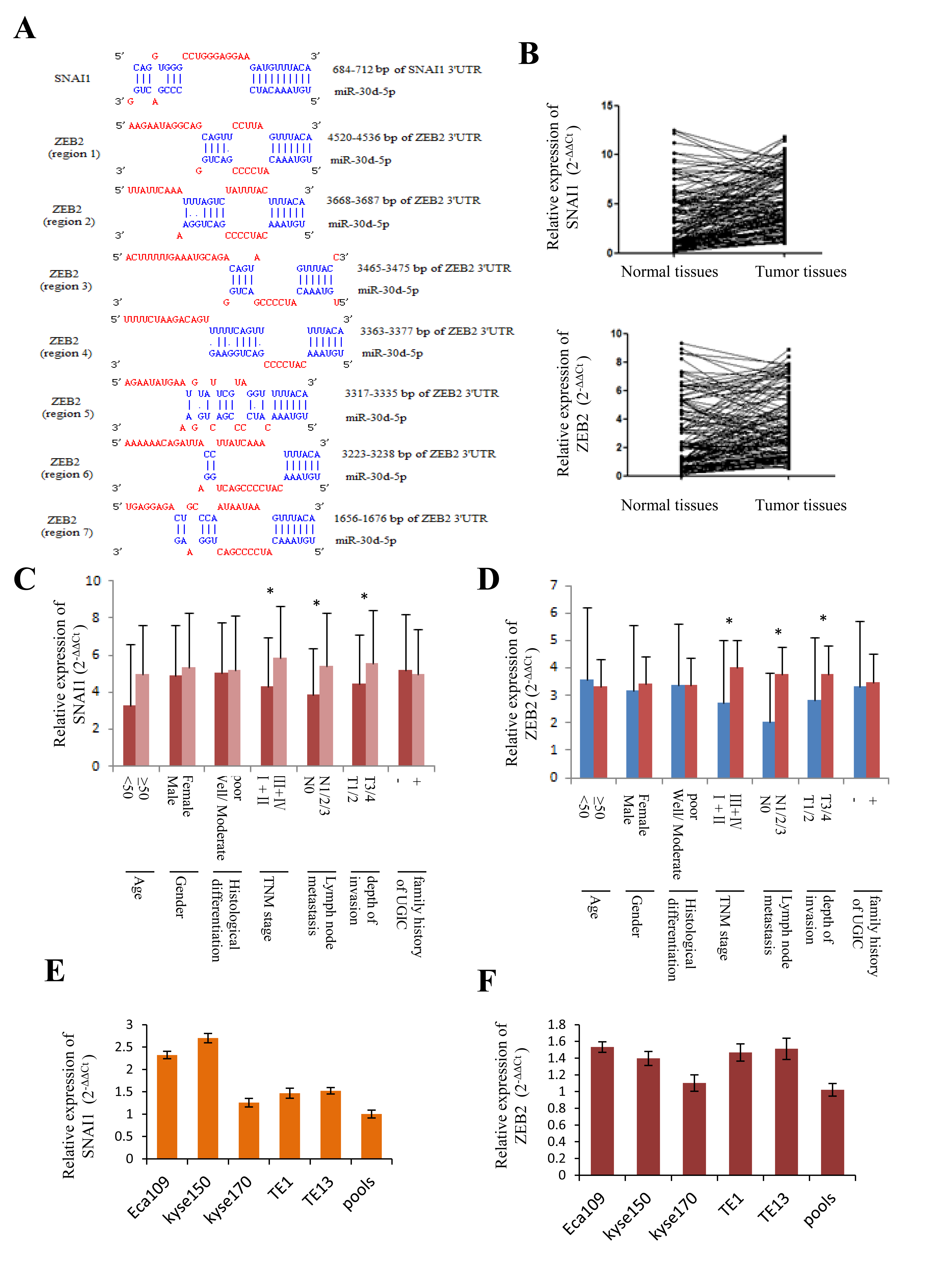

Supplement: Supplemental Material [file KBIE_A_2008759_SM0472.zip › supplementary/Fig.S2.tif]
